# Combined Impact of Neoadjuvant Therapy and Preoperative Cachexia in Patients Undergoing Pancreatoduodenectomy: Is There a “Double Jeopardy”? A National Cohort Study Investigating the Association with Short- and Long-Term Outcomes

**DOI:** 10.1245/s10434-025-18941-y

**Published:** 2026-01-05

**Authors:** Marcus Thomas Thor Roalsø, Celine Oanaes, Herish Garresori, Karin Hestnes Edland, Ingvild Dalen, Hanne Røland Hagland, Kjetil Søreide

**Affiliations:** 1 Department of Gastrointestinal Surgery, HPB Unit, Stavanger University Hospital, Stavanger, Norway; 2 Department of Quality and Health Technology, Faculty of Health Sciences, University of Stavanger, Stavanger, Norway; 3 Department of Oncology, Stavanger University Hospital, Stavanger, Norway; 4 Department of Research, Section of Biostatistics, Stavanger University Hospital, Stavanger, Norway; 5 Department of Chemistry, Bioscience and Environmental Engineering, University of Stavanger, Stavanger, Norway; 6 Department of Clinical Medicine, University of Bergen, Bergen, Norway

**Keywords:** Pancreatoduodenectomy, Cachexia, Neoadjuvant therapy, Textbook outcome, Survival analysis, Pancreatic cancer

## Abstract

**Background:**

Cachexia is associated with worse postoperative outcomes, but the added role of neoadjuvant therapy (NAT) is unclear. This study evaluated whether preoperative cachexia and NAT act as a “double jeopardy” after pancreatoduodenectomy.

**Patients and Methods:**

A nationwide observational cohort study was conducted using the Norwegian NORGAST registry (2016–2023). Adults undergoing pancreatoduodenectomy for malignancy were included. Cachexia was defined by consensus weight-loss criteria. Modified Poisson and Cox models (with a cachexia and NAT interaction term) estimated adjusted risk ratios (aRR) for textbook outcome (TO), prolonged length-of-stay (LOS), and adjusted hazard ratios (aHR) for overall survival.

**Results:**

Of 1424 patients undergoing pancreatoduodenectomy, cachexia was present in 588 (41.3%). Having cachexia was associated with higher TO (aRR 1.28, 95% CI 1.13–1.46) with effect modification by body mass index (BMI) (interaction *P* = 0.047). Patients with cachexia had a lower risk of prolonged LOS (aRR 0.64, 95% CI 0.51–0.80). Cachexia was not independently associated with overall survival (aHR 1.15, 95% CI 0.97–1.36). NAT was associated with a higher hazard of death (aHR 1.44, 95% CI 1.09–1.92), likely reflecting confounding by indication. No statistically significant interaction between cachexia and NAT was observed for TO (*P* = 0.277) or for survival (*P* = 0.863).

**Conclusions:**

Preoperative cachexia was associated with higher rates of TO. Higher TO was attributed to patients with overweight or obesity, to a shorter index stay, and more frequent transfers to a secondary facility, but not fewer complications. Cachexia was not associated with worse long-term survival, and a “double jeopardy” between cachexia and receiving NAT was not found.

**Electronic supplementary material:**

The online version of this article (10.1245/s10434-025-18941-y) contains supplementary material, which is available to authorized users.

Cachexia, a complex metabolic syndrome characterized by involuntary weight loss, muscle wasting, and systemic inflammation, affects 60% of patients with newly diagnosed pancreatic cancer.^1^ While pancreatoduodenectomy offers the potential for a cure for pancreatic head tumors, only 15–20% of patients are surgical candidates.^2^ Patients with cachexia often have compromised metabolic reserves, which may negatively impact postoperative recovery.^3,4^ However, available data on postoperative outcomes comparing patients with cachexia versus without remain conflicting.^5–7^ Some studies report higher complication rates and prolonged hospital stays in patients with cachexia, whereas others suggest fewer complications, possibly due to selective patient optimization before surgery.^4,5,8–11^ Nonetheless, patients with cachexia who develop postoperative complications may be particularly vulnerable and face an increased risk of postoperative morbidity and mortality.^4^ Increasingly, there is a focus on cachexia as a target for intervention, with novel drugs being investigated to counteract its effects in pancreatic cancer.^12–14^ Therefore, investigating the potential prognostic association of preoperative cachexia on surgical outcomes remains crucial.

Neoadjuvant therapy (NAT) is now a standard of care for patients with borderline or locally advanced pancreatic cancer.^15^ Its use is also widespread for resectable pancreatic cancer, despite conflicting data from randomized trials in the latter group.^16–18^ Furthermore, the interplay between systemic chemotherapy and a patient’s metabolic state is complex, as treatment can potentially exacerbate cachexia, while preexisting cachexia may influence treatment tolerance and response.^8,19^ Moreover, selection into NAT and subsequent selection for surgery complicate causal interpretation in observational cohorts. Better understanding these relationships could enhance patient selection, guide preoperative interventions, and inform efforts to improve outcomes after pancreatoduodenectomy.

This study aimed to evaluate the separate and combined associations of preoperative cachexia and NAT with postoperative outcomes after pancreatoduodenectomy, testing the “double jeopardy” hypothesis that cachexia modifies the association between NAT and postoperative outcomes such that patients with both exposures have higher postoperative risk.

## Patients and Methods

### Study Ethics

Data were sourced from the Norwegian Registry for Gastrointestinal Surgery (NORGAST), a nationwide quality register previously described.^20^ The registry holds a data storage license from the Norwegian Data Authority. The study received ethics exemption from the Regional Ethics Committee of Western Norway (REK Vest, #552496) and a confidentiality waiver from the Public Institute of Health (Helsedirektoratet, #24/43351-5).

### Study Design and Population

This nationwide observational cohort study utilized data from the NORGAST registry, encompassing all five Norwegian hospitals performing pancreatic surgery. Race and ethnicity are not collected in NORGAST, and therefore these variables were unavailable for this analysis. The study adhered to the Strengthening Reporting of Observational Studies in Epidemiology (STROBE) guidelines and the Reporting of studies Conducted using Observational Routinely collected health Data (RECORD) extension (Supplementary Fig. S1).

### Study Inclusion and Exclusion Criteria

Adult patients (≥ 18 years of age) undergoing pancreatoduodenectomy were included (Nordic Classification of Surgical Procedures [NCSP] code JLC30) between January 2016 and December 2023 and categorized on the basis of preoperative International Classification of Diseases, 10th Revision (ICD-10), diagnostic codes. “Malignancy” comprised C-codes (malignant neoplasms) or D-codes if NAT was administered. D-codes (in situ, benign, or uncertain neoplasms) without NAT were termed “other neoplasia,” including premalignant and undetermined neoplastic conditions. Final histopathological confirmation was unavailable in the registry. Patients with recorded hospital stays of ≤ 2 days were excluded as likely coding errors or atypical events, unless a postoperative death was recorded.

### Preoperative Cachexia

Patients were stratified by preoperative cachexia status, defined by established criteria^1^ as either weight loss ≥ 5% of body weight, or weight loss > 2% in patients with a body mass index (BMI) < 20 kg/m^2^. Weight loss was calculated from patient-reported weight 6 months before hospital admission and measured weight at admission for surgery.

### Comorbidities

Recorded comorbidities included diabetes (use of antidiabetic medication); severe cardiac disease (New York Heart Association class III or IV or the presence of arrhythmia requiring a cardiac implantable electronic device); and severe pulmonary disease (vital capacity < 60% or forced expiratory volume in 1 s < 50% of predicted value).

### Neoadjuvant Therapy

The NAT group comprised patients receiving chemotherapy within 3 months before surgery. Registry data lacked details on drug regimen or adjuvant therapy use, though the modified fluorouracil, leucovorin, irinotecan, and oxaliplatin (FOLFIRINOX) regimen was the most common treatment during the study period. NAT was routinely indicated for borderline or locally advanced pancreatic cancer, per National Comprehensive Cancer Network (NCCN) criteria,^21^ and the NAT group also included patients with resectable cancer, reflecting the concurrent NORPACT-1 trial, which was recruiting during the study period.^16^

### Outcome Measurement

Postoperative outcomes were classified according to the Expanded Accordion Classification system,^22^ with major complications defined as Accordion score ≥ 3. As the NORGAST registry lacks organ-specific complication data [e.g., postoperative pancreatic fistula (POPF)], Accordion score ≥ 3 served as a proxy for severe complications. This reflects the fact that severe complications often necessitate interventions (e.g., drain placement, reoperation, or management of organ failure) classified as Accordion score ≥ 3.

The TO definition was adapted from previous studies and adapted to fit the available data in the NORGAST registry.^23^ It required the absence of 30-day mortality, prolonged hospital stay (> 75th percentile, corresponding to 15 days for this cohort), severe complications (Accordion score ≥ 3), reoperation during index or a related readmission, and unplanned 30-day readmission. For surviving patients, TO status was considered undeterminable if any required data were missing. Prolonged length of stay (LOS) (> 15 days) was also analyzed as a standalone outcome.

Overall survival (OS) was defined as time from surgery to death from any cause. Patients were followed until the last follow-up date (February 2025), with data obtained via the National Institute of Public Health. Surviving patients were right-censored at the earlier of 5 years after surgery or the final follow-up date. The 30-day and 90-day mortality were defined as death from any cause within 30 and 90 days after surgery, respectively.

### Statistical Analysis

Statistical analyses were performed using R version 4.4.3 (R Foundation for Statistical Computing, Vienna, Austria). Packages utilized included mice (version 3.17.0)^24^ for imputation and gtsummary (version 2.0.3)^25^ for table generation.

Data are presented as medians with interquartile ranges (IQR) for continuous variables and as frequencies with percentages for categorical variables. Unadjusted group comparisons were performed using the Wilcoxon rank-sum test for continuous variables, Pearson’s chi-squared test, or Fisher’s exact test, as appropriate, for categorical variables.

Initial comparison revealed significant differences in key baseline variables between patients with complete (*N* = 1253) versus missing covariate data (*N* = 171) (Supplementary Table S1), prompting the use of multiple imputation by chained equations (MICE)^26^ under a missing-at-random (MAR) assumption for the primary multivariable analyses (*N* = 1424). A total of 20 imputed datasets (m = 20) were generated, with an imputation model including all outcomes, exposures, interaction terms, and covariates [age, sex, BMI category, American Society of Anesthesiologists (ASA) score, ECOG (Eastern Cooperative Oncology Group) score, diabetes, heart disease, lung disease, albumin, C-reactive protein (CRP), diagnosis type, and NAT status]. Each analysis model was fitted separately within each imputed dataset, and estimates (and standard errors) were combined across imputations using Rubin’s rules.^27^ Pooled adjusted risk ratios (aRRs) with 95% confidence intervals (CIs) are reported.

Multivariable modified Poisson regression with a log link and robust standard errors was used to directly estimate risk ratios (RRs), as the outcomes were not rare, and to assess associations with achieving the TO and with prolonged LOS. From the primary model, NAT-stratified adjusted probabilities of TO and corresponding risk differences (percentage points) were obtained via marginal standardization; stratum-specific RRs were estimated from the same model. For prolonged LOS (> 15 days, corresponding to the 75th percentile), the same model specification was applied to estimate stratum-specific adjusted RRs. The models included preoperative cachexia (yes/no) and NAT (yes/no) as main exposures. An interaction term between cachexia and NAT (cachexia × NAT) was included in the models to evaluate potential effect modification.

Models were adjusted for the a priori selected covariates age (continuous), sex (male versus female as reference), BMI category [underweight, normal (reference), overweight, obese], ASA score (≥ 3 versus 1–2 reference), ECOG score (≥ 2 versus 0–1 reference), diabetes mellitus (yes versus no reference), preoperative albumin (continuous), preoperative C-reactive protein (CRP) (continuous), and preoperative diagnosis type (“other neoplasia” versus “malignancy” reference).

Sensitivity analyses included a modified TO excluding prolonged LOS and separate models for each component outcome (severe complications, reoperation, 30-day readmission, and 30-day mortality). All analyses used the same modified Poisson specification and covariate adjustment as the primary TO analysis. Effect modification by BMI category was evaluated by adding a cachexia × BMI interaction term [BMI normal (reference) versus overweight/obese combined] to the modified TO model.

Unadjusted overall survival and median follow-up were estimated using the Kaplan–Meier and reverse Kaplan–Meier methods,^28^ respectively, with comparison between groups by the log-rank test. Analyses were performed for the overall cohort and for subgroups stratified by preoperative cachexia status, NAT status, preoperative diagnosis type, and combinations of cachexia and NAT status. As data for these stratification variables were complete, these descriptive survival analyses included all 1424 patients.

Cox proportional-hazards regression modeling^29^ was performed to investigate the independent and interactive effects of preoperative cachexia and NAT on long-term overall survival. The model used the 20 multiply imputed datasets (*N* = 1424) and included preoperative cachexia (yes/no), NAT (yes/no), and a cachexia × NAT interaction term. The covariate adjustment matched the primary model. The proportional-hazards assumption was examined using Schoenfeld residuals.^30^ Hazard ratios (HRs) with 95% CIs were reported, with estimates pooled across imputations as described previously.

Given the low number of events for 30-day (*N* = 21) and 90-day (*N* = 36) mortality in the full cohort (*N* = 1424), multivariable analyses were not performed for these short-term outcomes to avoid unreliable estimates due to sparse data across covariate strata. Unadjusted counts and percentages are reported, and between-group differences in mortality were evaluated using Pearson’s chi-squared test.

Statistical significance was defined as *P* < 0.050, and all tests were two-sided.

## Results

### Patient Characteristics

Of 1424 eligible patients, 588 (41.3%) met the definition of preoperative cachexia. Table 1 summarizes patient characteristics by cachexia status. Patients with cachexia were more frequently underweight or normal weight, more often received NAT, had higher rates of diabetes mellitus, and had worse ECOG performance status (Table 1).

**Table 1:** Preoperative characteristics of patients undergoing pancreatoduodenectomy, according to cachexia status

Characteristic	Overall *N* = 1424^1^	No cachexia *N* = 836^1^	Cachexia *N* = 588^1^	*P*-value^2^
Age (years)	69.8 (61.3, 74.9)	69.9 (60.9, 74.9)	69.6 (61.9, 75.0)	0.700
Sex				0.141
Female	653 (45.9%)	397 (47.5%)	256 (43.5%)	
Male	771 (54.1%)	439 (52.5%)	332 (56.5%)	
WHO BMI categories				< 0.001
Normal	677 (48.2%)	366 (44.5%)	311 (53.4%)	
Underweight	51 (3.6%)	18 (2.2%)	33 (5.7%)	
Overweight	496 (35.3%)	303 (36.9%)	193 (33.2%)	
Obese	180 (12.8%)	135 (16.4%)	45 (7.7%)	
Unknown	20	14	6	
Preoperative diagnosis				< 0.001
Malignancy	1306 (91.7%)	740 (88.5%)	566 (96.3%)	
Other neoplasia	118 (8.3%)	96 (11.5%)	22 (3.7%)	
ASA score				0.002
1–2	695 (48.8%)	437 (52.3%)	258 (43.9%)	
≥ 3	729 (51.2%)	399 (47.7%)	330 (56.1%)	
WHO ECOG score				< 0.001
0–1	1298 (93.7%)	771 (95.8%)	527 (90.9%)	
≥ 2	87 (6.3%)	34 (4.2%)	53 (9.1%)	
Unknown	39	31	8	
Neoadjuvant therapy	200 (14.0%)	96 (11.5%)	104 (17.7%)	< 0.001
Diabetes mellitus	245 (17.2%)	118 (14.1%)	127 (21.6%)	< 0.001
Heart disease	35 (2.5%)	24 (2.9%)	11 (1.9%)	0.230
Lung disease	29 (2.0%)	21 (2.5%)	8 (1.4%)	0.130
Albumin (g/L)	40.0 (36.0, 43.0)	41.0 (37.0, 43.0)	40.0 (36.0, 42.0)	< 0.001
Unknown	45	38	7	
CRP (mg/L)	5.0 (2.0, 13.0)	5.0 (2.0, 12.0)	6.0 (2.0, 14.0)	0.044
Unknown	107	88	19	

^1^Median (Q1, Q3); *n* (%)

^2^Wilcoxon rank sum test; Pearson’s chi-squared test

Preoperative diagnosis: clinical and imaging assessment; albumin/CRP: latest values within 3 weeks (21 days) preop; ECOG: mean during latest 3 weeks predating admission; BMI: recorded at the preoperative evaluation (post-NAT for recipients); comorbidities: medical history

*BMI* body mass index, *ASA* American Society of Anesthesiologists, *WHO* World Health Organization, *ECOG* Eastern Cooperative Oncology Group, *CRP* C-reactive protein

Diagnostic codes indicated malignancy in 91.7% (*N* = 1306) of patients and “other neoplasia” in 8.3% (*N* = 118). The “other neoplasia” group was heterogeneous (details in Supplementary Table S2).

### Postoperative Short-Term Outcomes

Unadjusted short-term postoperative outcomes are detailed in Supplementary Table S3. Patients with cachexia had a shorter median hospital stay (8 days versus 10 days, *P <* 0.001) and a higher rate of achieving the TO (52.0% versus 41.1%, *P* < 0.001) compared with non-cachectic patients. Cachexia was associated with a higher incidence of relaparotomy upon readmission (20.6% of readmitted patients with cachexia versus 9.8% of readmitted non-cachectic patients, *P* = 0.014).

There were no significant unadjusted differences in rates of severe complications (Accordion ≥ 3), overall readmission, reoperation, 30-day mortality, or 90-day mortality between groups.

Discharge destinations differed significantly (*P* < 0.001). Patients with cachexia were less often discharged home (32% versus 46%) and more frequently transferred to another hospital facility (64% versus 50%). Discharge to nursing facilities was similar for both patients with cachexia and without (3.4% versus 3.5%).

### Multivariable Analysis of Textbook Outcome and Prolonged Length of Stay

Multivariable regression (Table 2A and Supplementary Fig. S2) showed that preoperative cachexia was associated with a higher likelihood of TO (aRR 1.28, 95% CI 1.13–1.46; *P* < 0.001). Male sex, ECOG score ≥ 2, and a preoperative diagnosis of neoplasia (versus malignancy) were associated with lower TO. To aid interpretation, NAT-stratified marginal risks are presented in Table 2B. Among patients without NAT, the adjusted probability of TO was 39.9% (95% CI 36.3–43.4) for those with no cachexia versus 51.2% (46.6–55.8) for those with cachexia, an RD of +11.3 pp (95% CI +5.5 to +17.1) and RR 1.28 (1.13–1.46). Among those with NAT, risks were 44.3% (34.7–53.9) versus 48.0% (38.8–57.1), RD +3.7 pp (−9.6 to +16.9) and RR 1.08 (0.81–1.44). The cachexia × NAT interaction was not statistically significant (*P* = 0.277). Estimates were similar in a sensitivity model excluding ECOG (Table 2C).

**Table 2:** Multivariable Poisson regression analysis using multiple imputation for factors associated with achieving the textbook outcome

(A) Adjusted risk ratios		
Variable	Adjusted RR (95% CI)	*P*
Primary exposures		
Cachexia: Yes (Ref: No)	1.28 (1.13–1.46)	< 0.001
Neoadjuvant therapy: Yes (Ref: No)	1.11 (0.88–1.40)	0.373
Cachexia × NAT: P for interaction	–	0.277
Demographics		
Age (per 10 years)	0.98 (0.92–1.04)	0.419
Sex: Male (Ref: Female)	0.77 (0.68–0.86)	< 0.001
Anthropometrics		
BMI: Normal (Ref)	1.00 (Ref)	–
BMI: Underweight	0.97 (0.74–1.27)	0.808
BMI: Overweight	0.87 (0.77–1.00)	0.045
BMI: Obese	0.96 (0.80–1.15)	0.634
Comorbidity		
Diabetes: Yes (Ref: No)	1.11 (0.96–1.29)	0.158
Heart disease: Yes (Ref: No)	1.00 (0.64–1.55)	0.991
Lung disease: Yes (Ref: No)	0.85 (0.49–1.46)	0.557
Laboratory		
Albumin (g/L)	1.00 (0.98–1.01)	0.497
CRP (mg/L)	1.00 (1.00–1.00)	0.635
Disease		
Diagnosis: Other neoplasia (Ref: Malignancy)	0.71 (0.53–0.94)	0.015

(B) NAT-stratified marginal risks with risk differences and risk ratios				
NAT	Adjusted risk, no cachexia % (95% CI)	Adjusted risk, Cachexia % (95% CI)	RD, pp (95% CI)	RR (95% CI)
No NAT	39.9 (36.3–43.4)	51.2 (46.6–55.8)	+11.3 (+5.5 to +17.1)	1.28 (1.13–1.46)
Yes NAT	44.3 (34.7–53.9)	48.0 (38.8–57.1)	+3.7 (−9.6 to +16.9)	1.08 (0.81–1.44)

(C) Sensitivity analysis excluding ECOG		
Variable	Adjusted RR (95% CI)	P
Cachexia: Yes (Ref: No)	1.26 (1.11–1.43)	**< 0.001**
Neoadjuvant therapy: Yes (Ref: No)	1.11 (0.88–1.40)	0.372
Cachexia × NAT (interaction)	0.86 (0.63–1.17)	0.335

Adjusted risk ratios from modified Poisson regression (robust SEs) with multiple imputation (m = 20). RR > 1 denotes higher probability of achieving the textbook outcome; RR < 1 denotes lower probability. Reference groups shown

*aRR* adjusted risk ratio, *CI* confidence interval, *NAT* neoadjuvant therapy, *BMI* body mass index, *ASA* American Society of Anesthesiologists, *WHO* World Health Organization, *ECOG* Eastern Cooperative Oncology Group, *CRP* C-reactive protein

Values are marginal predictions from the panel A model (response scale for risks and RD; link scale for RR); contrasts are cachexia/no cachexia within NAT strata. A positive RD indicates a higher adjusted probability of achieving TO in cachexia versus no cachexia

*RD* risk difference, *RR* risk ratio, *pp* percentage points

Sensitivity model excludes ECOG.

**Fig. 1:**
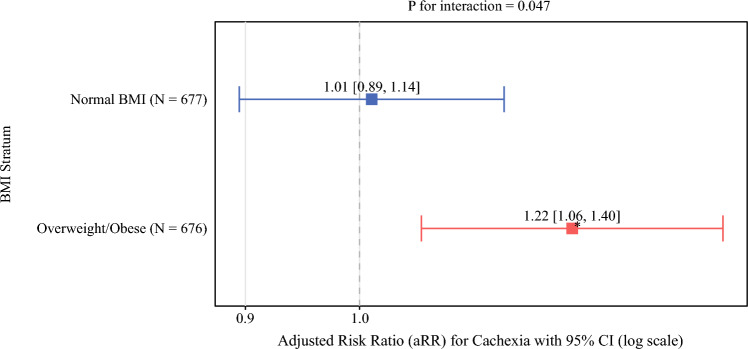
Effect modification by BMI on the association between cachexia and textbook outcome; forest plot of adjusted risk ratios (aRRs) for the association between cachexia and achieving the modified textbook outcome, stratified by BMI category; estimates derived from multivariable modified Poisson regression with robust standard errors and multiple imputation (m = 20); squares represent the pooled aRRs, horizontal lines the 95% confidence intervals, and vertical dashed line the null value (aRR = 1.0); interaction *P* = 0.047; *P < 0.05

In exploratory analyses, the association between cachexia and the modified TO varied by BMI (interaction *P* = 0.047; Fig. 1). Among patients with normal BMI, cachexia was not associated with achieving the modified TO (aRR 1.01, 95% CI 0.89–1.14). However, in overweight and obese patients, cachexia was associated with a higher probability of achieving TO (aRR 1.22, 95% CI 1.06–1.40). Stratification by neoadjuvant therapy status was not conducted because of insufficient sample size.

A sensitivity analysis excluding LOS from the TO definition showed that cachexia remained associated with higher probability of achieving this outcome among patients without NAT (aRR 1.13, 95% CI 1.03–1.25, *P* = 0.013), though the association was attenuated. Among patients without cachexia, the NAT effect was not significant (aRR 1.03, 95% CI 0.85–1.23, *P* = 0.791), and the cachexia × NAT interaction test was not statistically significant (*P* = 0.215). Factors associated with lower probability of the modified TO were similar to the primary TO model (Supplementary Table S4). Analysis of the individual component outcomes showed a significantly lower probability of prolonged LOS (> 75th percentile) among patients with cachexia (aRR 0.64, 95% CI 0.51–0.80, *P* < 0.001), whereas there were no significant associations with severe complications, reoperation, 30-day readmission, or 30-day mortality (all *P* > 0.05, Supplementary Fig. S3).

For prolonged LOS (> 15 days, 75th percentile), cachexia was independently associated with significantly lower risk (pooled aRR 0.64, 95% CI 0.51–0.80, *P* < 0.001) (Supplementary Table S5). Male sex, obesity, ECOG score (≥ 2), and “other neoplasia” diagnosis were associated with higher risk of prolonged LOS (Supplementary Table S5). The cachexia × NAT interaction for prolonged LOS was not statistically significant (*P* = 0.638). Complete case analysis gave similar results (Supplementary Tables S6–S7).

### Survival Analysis

**Fig. 2:**
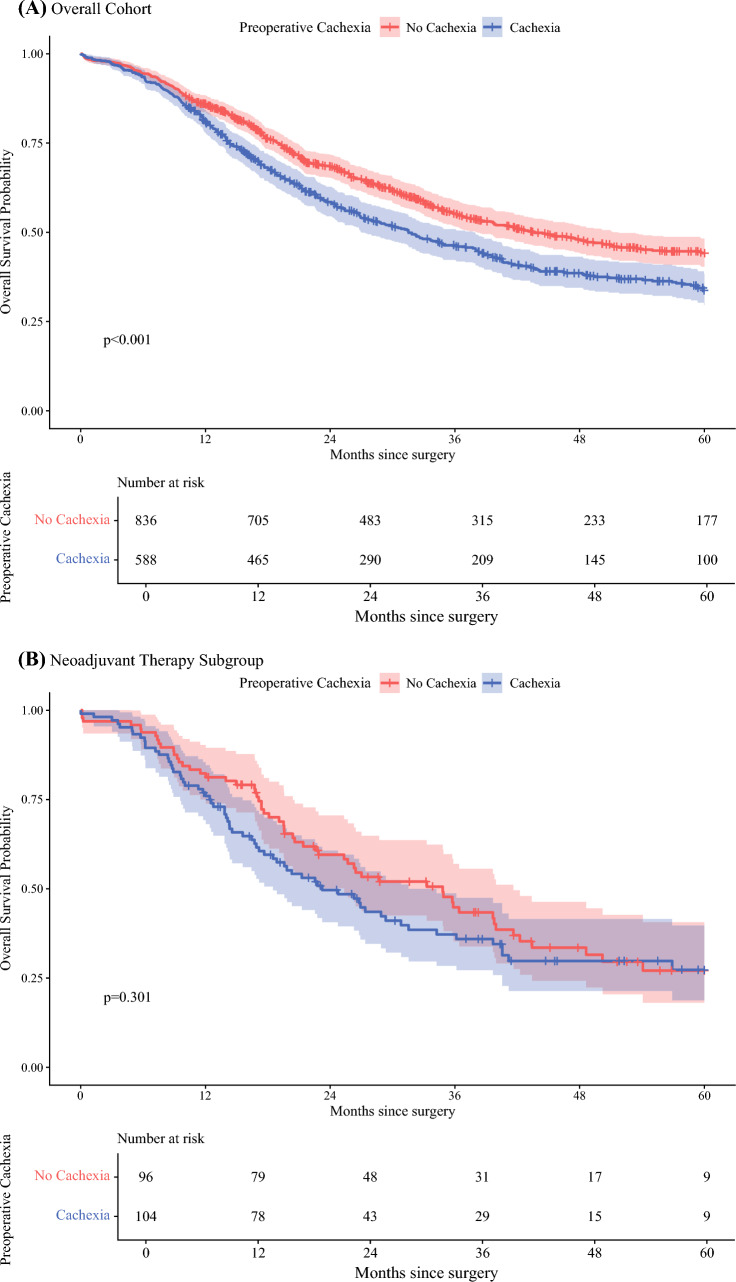

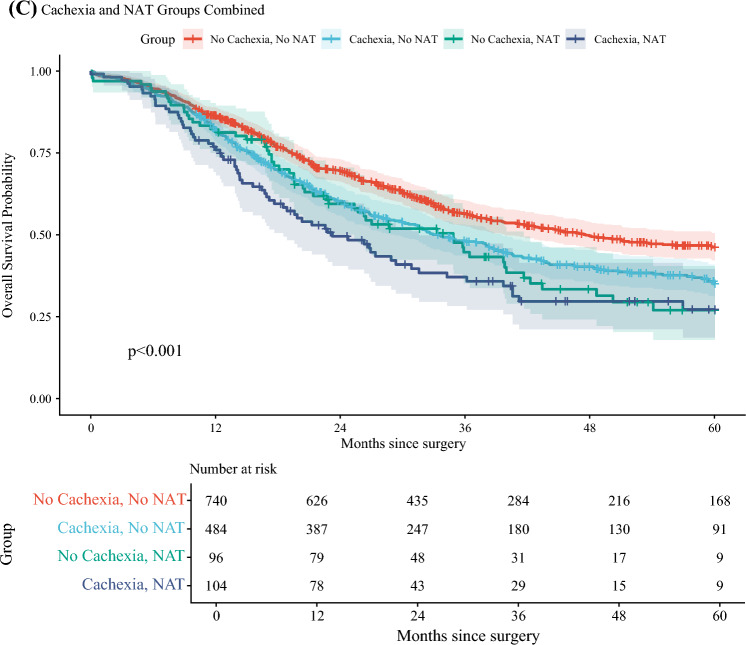
Kaplan–Meier survival estimates; **A** overall cohort; survival stratified by preoperative cachexia in the overall cohort (*N* = 1424); **B** neoadjuvant therapy subgroup; survival stratified by preoperative cachexia in patients receiving neoadjuvant therapy (*N* = 200); **C** cachexia and NAT groups combined; overall survival stratified by combined preoperative cachexia and neoadjuvant therapy (NAT) status: no cachexia/no NAT; cachexia/no NAT; no cachexia/with NAT; cachexia/with NAT; *P*-value from log-rank test comparing the four groups

**Fig. 3:**
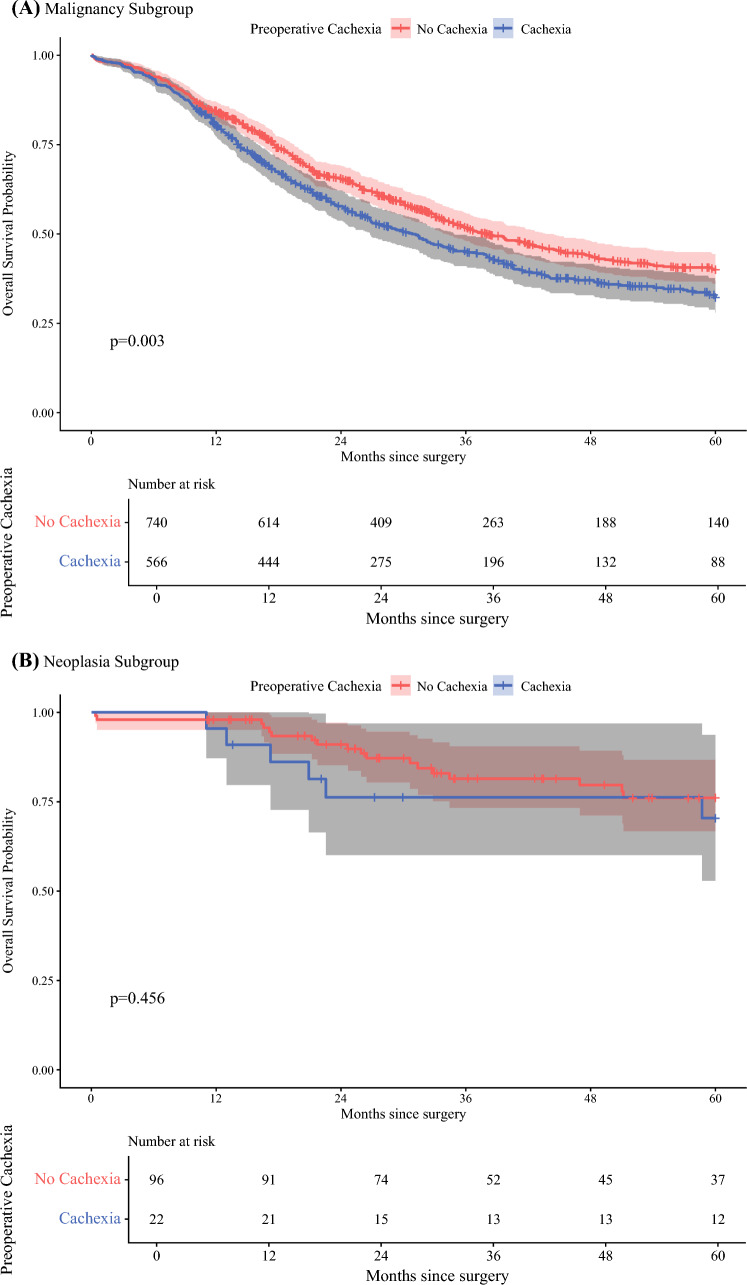
Kaplan–Meier survival estimates stratified by preoperative cachexia in patients with **A** preoperative malignancy, survival stratified by preoperative cachexia in patients with preoperative diagnosis of malignancy (*N* = 1306); and **B** preoperative neoplasia diagnosis, survival stratified by preoperative cachexia in patients with preoperative diagnosis of neoplasia (*N* = 118)

The association of preoperative cachexia with long-term survival is presented in Fig. 2 and Fig. 3. During the study period, 762 (53.5%) deaths were recorded. The median follow-up for the overall cohort was 59.2 months (IQR 32.5–80.8). Among patients alive at the end of the study period, the median follow-up was 44.9 months (IQR 24.0–73.0), whereas the median time to event for deceased patients was 18.2 months (IQR 10.3–30.8). In the overall cohort (*N* = 1424), patients with preoperative cachexia had worse unadjusted overall survival compared with non-cachectic patients (log-rank *P* < 0.0001, Fig. 2A). This survival disadvantage associated with cachexia was predominantly observed among patients with a preoperative diagnosis of malignancy (*N* = 1306), wherein cachexia was associated with poorer unadjusted survival (log-rank *P* = 0.0035, Fig. 3A). Conversely, in the smaller subgroup of patients receiving neoadjuvant therapy (*N* = 200), there was no statistically significant difference in overall survival between patients with cachexia and without (log-rank *P* = 0.301, Fig. 2B). Similarly, among patients with a preoperative diagnosis of “other neoplasia” (*N* = 118), cachexia was not significantly associated with a difference in overall survival, although event rates were low in this subgroup (log-rank *P* = 0.456, Fig. 3B).

### Multivariable Analysis of Long-Term Overall Survival

The results of the multivariable Cox proportional-hazards regression analysis for long-term overall survival are detailed in Table 3 and Supplementary Fig. S4.

**Table 3:** Multivariable Cox proportional-hazards regression analysis of factors associated with long-term overall survival

Characteristic	aHR	95% CI	*P*-value
Preoperative cachexia			
No cachexia	–	–	
Cachexia	1.15	0.97, 1.36	0.098
Neoadjuvant therapy (NAT)			
No	–	–	
Yes	1.44	1.09, 1.92	0.011
Age (years)	1.03	1.02, 1.04	< 0.001
Sex			
Female	–	–	
Male	1.09	0.94, 1.27	0.244
BMI category			
Normal	–	–	
Underweight	1.71	1.19, 2.46	0.004
Overweight	1.09	0.92, 1.28	0.328
Obese	1.11	0.87, 1.42	0.391
ASA score			
1–2	–	–	
≥ 3	1.14	0.97, 1.34	0.101
WHO/ECOG score			
0–1	–	–	
≥ 2	1.60	1.22, 2.10	< 0.001
Diabetes mellitus			
No	–	–	
Yes	1.15	0.96, 1.39	0.137
Heart disease			
No	–	–	
Yes	0.67	0.38, 1.16	0.152
Lung disease			
No	–	–	
Yes	1.12	0.67, 1.85	0.668
Preop albumin (g/L)	0.97	0.96, 0.99	0.002
Preop CRP (mg/L)	1.01	1.00, 1.01	< 0.001
Diagnosis type			
Malignancy	–	–	
Other neoplasia	0.34	0.23, 0.52	< 0.001
Cachexia × NAT interaction			
Cachexia × Yes	1.04	0.70, 1.53	0.863

Results based on 20 multiply imputed datasets

*aHR* adjusted hazard ratio, *CI* confidence interval, *NAT* neoadjuvant therapy, *BMI* body mass index, *ASA* American Society of Anesthesiologists, *WHO* World Health Organization, *ECOG* Eastern Cooperative Oncology Group, *CRP* C-reactive protein

After adjusting for prognostic covariates, preoperative cachexia was not significantly associated with overall survival (aHR 1.15, 95% CI 0.97–1.36, *P* = 0.098). However, receiving neoadjuvant therapy (NAT) was associated with a higher hazard of death (aHR 1.44, 95% CI 1.09–1.92, *P* = 0.011) compared with not receiving NAT. The interaction term between preoperative cachexia and NAT (cachexia × NAT) was not statistically significant (aHR 1.04, 95% CI 0.70–1.53, *P* = 0.863).

## Discussion

This study found no evidence that preoperative cachexia combined with NAT worsens postoperative outcomes or survival after pancreatoduodenectomy. Nevertheless, some associations were identified that may warrant further in-depth investigations. For one, the presence of cachexia was paradoxically associated with a higher probability of achieving TO, an effect that appeared confined to patients with overweight or obesity. However, the favorable association with achieving TO appears to be attributed to shorter LOS for patients with cachexia, rather than a reduction in severe complications. Nevertheless, this shorter LOS did not necessarily indicate faster clinical recovery, as a short LOS was strongly related to earlier transfers to other healthcare facilities instead of home. Indeed, complication rates were similar between groups, with a sensitivity analysis showing that when LOS was removed from the TO definition, the association with cachexia was attenuated. The findings do not indicate that preoperative cachexia should be viewed as a standalone contraindication to NAT or pancreatoduodenectomy, but a selection bias of operated patients is present in the current series. This suggests that index hospital LOS may be influenced by system-level logistics and discharge pathways described in previous studies,^23,31,32^ and should be considered when comparing studies using similar composite endpoints.

The paradoxical association between cachexia and TO varied with BMI, indicating effect modification. This pattern is consistent with the “obesity paradox” in critical illness, in which higher BMI has been associated with better outcomes^33^ . This pattern may relate to greater metabolic reserves, as patients with higher BMI, even when cachectic, may better tolerate the catabolic stress of pancreatoduodenectomy. This interaction may help reconcile conflicting reports on the prognostic significance of cachexia^9,19,34^ , and underscores the need for more nuanced, body-composition-based preoperative assessments.

For long-term survival, the prognostic association of cachexia was similarly attenuated after multivariable adjustment. This suggests that the relationship is largely captured by more objective markers of patient physiology and functional status, as lower preoperative albumin, higher CRP, and lower ECOG score remained independent predictors of mortality after adjusting for covariates. The controversial role and definitions used for cachexia align with previous studies advocating for a more objective assessment beyond simple weight loss, such as imaging-based body composition changes,^9,34^ which were not available in the current cohort. A universally agreed-upon definition and widespread clinical use of cachexia screening for intervention are still lacking but may increase in importance as drugs targeting cachexia become available.

The current study does not support the notion that NAT is linked to an additional short-term burden or long-term survival detriment for patients with cachexia, as the interaction term was not significant in either model investigated for this. Importantly, the absence of a “double jeopardy” effect suggests that clinicians should not withhold NAT from patients with cachexia solely out of concerns for compounded surgical risk. Instead, these patients may benefit from targeted prehabilitation during the neoadjuvant window.^35^ Nonetheless, the absence of a “double jeopardy” effect is likely a product of selection bias. Patients with cachexia who complete both NAT and major surgery may represent a more physiologically resilient subgroup, susceptible to survivorship bias.^36,37^ Similarly, the finding that NAT was independently associated with poorer long-term survival should not be interpreted as evidence of treatment harm. More plausibly, this reflects confounding by indication, where patients with borderline or locally advanced disease and an inherently worse prognosis are selected for neoadjuvant therapy.^18,38^ The poorer survival may hence reflect an a priori more advanced disease stage to indicate start of NAT.

This study has limitations that warrant discussion. One inherent limitation of a registry-based cohort is the lack of granular data for certain variables, such as specific complications including postoperative pancreatic fistulas. However, complications were ascertained using the Accordion score, providing standardized, nationwide capture of clinically relevant events. Further, the definition of cachexia relied on patient-reported weight loss, which lacks the objectivity of imaging-based sarcopenia assessments, a known predictor of clinical outcomes.^3,11^ Nonetheless, a previous validation study in a similar Norwegian population has demonstrated substantial agreement between self-reported and measured anthropometrics.^39^ Furthermore, the definition used in this study is based on established international criteria and has been widely reported across other studies. The observed prevalence of 41.3% aligns with findings from similar surgical cohorts (40.5%) and also falls within the wider prevalence range of 21.3–63% reported in the literature.^40,41^ This variation likely reflects heterogeneity between patient cohorts and differences in cachexia definitions, with the estimate in this study reflecting the unselected nature of a nationwide registry. Also, image-based sarcopenia lacks a universally accepted definition, with many proposed definitions available, and even an image-based definition would introduce variation in comparability. Notably, the definition of cachexia used in the current study can be reproduced in similar datasets. Furthermore, the registry lacks detailed data on specific neoadjuvant therapies, granular longitudinal weight measurements during NAT, and specific tumor classifications (i.e., stages), which could influence both selection for surgery and outcomes.

Despite some shortcomings, a key strength of the study is the nationwide, complete cohort of unselected patients who underwent pancreatoduodenectomy within a universal healthcare system without competing private practice. This provides a robust, population-based assessment with uniform access to care and consensus-based clinical practices, thereby enhancing the generalizability to a real-world surgical setting.

In conclusion, the association of preoperative cachexia and postoperative outcomes in patients undergoing pancreatoduodenectomy is complex and conditional on a patient’s underlying body composition. In this cohort, the favorable finding for TO observed only in patients with overweight and obesity and cachexia appeared to relate to a shorter LOS and did not necessarily reflect a superior clinical recovery. While cachexia is an important clinical sign, its prognostic value for both short- and long-term survival may be better captured by objective markers of a patient’s metabolic, inflammatory, and functional state. Future research should prioritize the development of more meaningful short-term outcome measures and explore body-composition-based risk stratification to guide targeted interventions for this vulnerable patient population.

## Electronic supplementary material

Supplementary material 1

Supplementary material 2

## Data Availability

Deidentified registry data are available upon formal application to the NORGAST Registry through the National Health Data Service (Helsedataservice).
