# Supplementary material for: Combined Impact of Neoadjuvant Therapy and Preoperative Cachexia in Patients Undergoing Pancreatoduodenectomy: Is There a “Double Jeopardy”? A National Cohort Study Investigating the Association with Short- and Long-Term Outcomes
Source: Ann Surg Oncol. 2026 Jan 5;33(4):3563–75. doi: 10.1245/s10434-025-18941-y (PMC12982233; doi:10.1245/s10434-025-18941-y)
Supplement: Supplementary file 1 — Supplementary material 1 [file 10434_2025_18941_MOESM1_ESM.docx]

**The RECORD statement – checklist of items, extended from the STROBE statement, that should be reported in observational studies using routinely collected health data.**

|  | **Item No.** | **STROBE items** | **Location in manuscript where items are reported** | **RECORD items** | **Location in manuscript where items are reported** |
| --- | --- | --- | --- | --- | --- |
| **Title and abstract** | | | | | |
|  | 1 | (a) Indicate the study’s design with a commonly used term in the title or the abstract (b) Provide in the abstract an informative and balanced summary of what was done and what was found | (a) “**Methods:** A nationwide observational cohort study was conducted using the Norwegian NORGAST registry (2015–2024). … models (with a cachexia × NAT interaction term) estimated adjusted risk ratios (aRR…)”  (b) "**Background**: Cachexia is associated with worse postoperative outcomes... Methods: A nationwide observational cohort study was conducted... **Results**: Of 1,424 patients...cachexia was present in 588 (41.3%). Having cachexia was associated with higher TO (aRR 1.28...). **Conclusion**: Preoperative cachexia was associated with higher rates of TO... attributed to... a shorter index stay... but not fewer complications." | RECORD 1.1: The type of data used should be specified in the title or abstract. When possible, the name of the databases used should be included.  RECORD 1.2: If applicable, the geographic region and timeframe within which the study took place should be reported in the title or abstract.  RECORD 1.3: If linkage between databases was conducted for the study, this should be clearly stated in the title or abstract. | **(**1.1) **Title:** "A National Cohort Study..."  **Abstract:** "A nationwide observational cohort study was conducted using the Norwegian NORGAST registry..."  (1.2) **Abstract:** "Norwegian NORGAST registry (2016–2023)."  **(**1.3) **Abstract:** "Patients were followed until the last follow-up date... with data obtained via the National Institute of Public Health." |
| **Introduction** | | | | | |
| Background rationale | 2 | Explain the scientific background and rationale for the investigation being reported | “Cachexia, a complex metabolic syndrome characterized by involuntary weight loss… is associated with poor outcomes… Understanding the separate and combined effects of preoperative cachexia on surgical outcomes remains crucial.” |  |  |
| Objectives | 3 | State specific objectives, including any prespecified hypotheses | “**This study aimed** to evaluate the separate and combined associations of preoperative cachexia and neoadjuvant therapy (NAT) with short- and long-term outcomes… and to test whether patients with both exposures have higher postoperative risk.” |  |  |
| **Methods** | | | | | |
| Study Design | 4 | Present key elements of study design early in the paper | “This **nationwide observational cohort** study utilized data from the Norwegian NORGAST registry… The study adhered to the STROBE guidelines (Supplementary Figure S1).” |  |  |
| Setting | 5 | Describe the setting, locations, and relevant dates, including periods of recruitment, exposure, follow-up, and data collection | “Adult patients (≥18 years of age) undergoing pancreatoduodenectomy between January 2016 and December 2023 were included. Data were sourced from The Norwegian Registry for Gastrointestinal Surgery (NORGAST), which includes all five Norwegian hospitals performing pancreatic surgery.” |  |  |
| Participants | 6 | *(a) Cohort study* - Give the eligibility criteria, and the sources and methods of selection of participants. Describe methods of follow-up  *Case-control study* - Give the eligibility criteria, and the sources and methods of case ascertainment and control selection. Give the rationale for the choice of cases and controls  *Cross-sectional study* - Give the eligibility criteria, and the sources and methods of selection of participants  *(b) Cohort study* - For matched studies, give matching criteria and number of exposed and unexposed  *Case-control study* - For matched studies, give matching criteria and the number of controls per case | (a) “Adult patients (≥18 years)… undergoing pancreatoduodenectomy… were included; patients with missing essential variables or atypical events were excluded…”  “**Overall survival (OS)** was defined as time from surgery to death from any cause… **30-day** and **90-day** mortality were defined as death within 30 and 90 days after surgery, respectively.”  (b) N/A | RECORD 6.1: The methods of study population selection (such as codes or algorithms used to identify subjects) should be listed in detail. If this is not possible, an explanation should be provided.  RECORD 6.2: Any validation studies of the codes or algorithms used to select the population should be referenced. If validation was conducted for this study and not published elsewhere, detailed methods and results should be provided.  RECORD 6.3: If the study involved linkage of databases, consider use of a flow diagram or other graphical display to demonstrate the data linkage process, including the number of individuals with linked data at each stage. | (6.1) **Methods 2.3:** "Adult patients... undergoing pancreatoduodenectomy were included (Nordic Classification of Surgical Procedures [NCSP] code JLC30)... and categorized based on preoperative ICD-10 diagnostic codes."  (6.2) **Methods 2.1:** Refers to previous NORGAST validation/description.  **Discussion:** Discussion of self-reported weight validation (Skeie et al. reference) and Accordion score standardization.  (6.3) **Methods 2.7:** Linkage to National Institute of Public Health for survival.  **Supplementary Figure S1:** STROBE Flow diagram |
| Variables | 7 | Clearly define all outcomes, exposures, predictors, potential confounders, and effect modifiers. Give diagnostic criteria, if applicable. | “Patients were stratified by **preoperative cachexia** status, defined by… the difference between the usual body weight before hospital admission and the measured weight at admission for surgery.”  “The **NAT group** comprised patients receiving chemotherapy and/or radiotherapy before surgery…”  “Primary endpoints included **textbook outcome (TO)**, **prolonged length of stay (LOS)**, **30-/90-day mortality**, and **overall survival**.” | RECORD 7.1: A complete list of codes and algorithms used to classify exposures, outcomes, confounders, and effect modifiers should be provided. If these cannot be reported, an explanation should be provided. | **Methods 2.3:** Definitions of Malignancy (C-codes) vs Other Neoplasia (D-codes).  **Methods 2.4:** Cachexia definition.  **Supplementary Table S2:** List of specific ICD-10 codes. |
| Data sources/ measurement | 8 | For each variable of interest, give sources of data and details of methods of assessment (measurement).  Describe comparability of assessment methods if there is more than one group | Data were sourced from **The Norwegian Registry for Gastrointestinal Surgery (NORGAST)**… approvals obtained from…”  “Cachexia was defined/ascertained as…” |  |  |
| Bias | 9 | Describe any efforts to address potential sources of bias | “Models were adjusted for the a priori…” |  |  |
| Study size | 10 | Explain how the study size was arrived at | “All adult patients undergoing pancreatoduodenectomy between January 2016 and December 2023 in the NORGAST registry were considered. The final study cohort eligible for analysis consisted of 1,424 patients.” |  |  |
| Quantitative variables | 11 | Explain how quantitative variables were handled in the analyses. If applicable, describe which groupings were chosen, and why | “Data are presented as **medians (IQR)**… Continuous and categorical variables were compared using **Wilcoxon rank-sum** and **Pearson’s chi-square/Fisher’s exact** tests, respectively.” |  |  |
| Statistical methods | 12 | (a) Describe all statistical methods, including those used to control for confounding  (b) Describe any methods used to examine subgroups and interactions  (c) Explain how missing data were addressed  (d) *Cohort study* - If applicable, explain how loss to follow-up was addressed  *Case-control study* - If applicable, explain how matching of cases and controls was addressed  *Cross-sectional study* - If applicable, describe analytical methods taking account of sampling strategy  (e) Describe any sensitivity analyses | (a) “**Multivariable modified Poisson regression** with a log link… estimated aRRs with 95% CIs… **Cox proportional-hazards** regression for OS… **Models were adjusted** for a priori covariates (age, sex, ASA, BMI categories, diagnosis type…).”  (b) “An **interaction** between cachexia and **BMI** was included in the models to evaluate potential **effect modification**.”  (c) “Analyses were performed in **R**… using **multiple imputation**…; estimates were **pooled across imputations** as described previously.”  (d) N/A  (e) “**Sensitivity analyses** included a **modified TO** excluding prolonged LOS… and alternative BMI specification…”  “A **sensitivity analysis** excluding LOS from the TO definition showed…” |  |  |
| Data access and cleaning methods |  | .. |  | RECORD 12.1: Authors should describe the extent to which the investigators had access to the database population used to create the study population.  RECORD 12.2: Authors should provide information on the data cleaning methods used in the study. | (12.1) **Section 7 (Disclaimer):** "Data... has been used... interpretation... sole responsibility of the authors."  **Data Availability Statement:** "De-identified registry data are available upon formal application..."  (12.2) **Methods 2.3:** "Patients with recorded hospital stays of ≤2 days were excluded as likely coding errors..."  **Methods 2.8:** Multiple Imputation (MICE) used for missing data. |
| Linkage |  | .. |  | RECORD 12.3: State whether the study included person-level, institutional-level, or other data linkage across two or more databases. The methods of linkage and methods of linkage quality evaluation should be provided. | **Methods 2.7:** "Patients were followed... with data obtained via the National Institute of Public Health."  **Methods 2.1:** Registry holds data storage license from Norwegian Data Authority. |
| **Results** | | | | | |
| Participants | 13 | (a) Report the numbers of individuals at each stage of the study (*e.g.*, numbers potentially eligible, examined for eligibility, confirmed eligible, included in the study, completing follow-up, and analysed)  (b) Give reasons for non-participation at each stage.  (c) Consider use of a flow diagram | (a) “**Of 1,424 eligible patients**, 588 (41.3%) met the definition of preoperative cachexia… **Table 1** summarizes patient characteristics…”  (b) “Patients with **missing essential variables** or **atypical events** (unless postoperative death recorded) were **excluded**…”  (c) “**Figure S1.** STROBE Diagram” | RECORD 13.1: Describe in detail the selection of the persons included in the study (*i.e.,* study population selection) including filtering based on data quality, data availability and linkage. The selection of included persons can be described in the text and/or by means of the study flow diagram. | **Results 3.1:** "Of 1,424 eligible patients..."  **Supplementary Figure S1:** STROBE Diagram detailing exclusions (e.g., non-PD procedures, non-C/D codes). |
| Descriptive data | 14 | (a) Give characteristics of study participants (*e.g.*, demographic, clinical, social) and information on exposures and potential confounders  (b) Indicate the number of participants with missing data for each variable of interest  (c) *Cohort study* - summarise follow-up time (*e.g.*, average and total amount) | (a) “**Table 1** summarizes patient characteristics by cachexia status…”  (b) “**Table S1.** Comparison of Complete Cases vs Cases with Missing Covariates”  (c) “**OS** was defined as time from surgery to death… **30-/90-day mortality** defined as…” |  |  |
| Outcome data | 15 | *Cohort study* - Report numbers of outcome events or summary measures over time  *Case-control study* - Report numbers in each exposure category, or summary measures of exposure  *Cross-sectional study* - Report numbers of outcome events or summary measures | “For **prolonged LOS (>15 days)**… cachexia was associated with higher risk…; Unadjusted and adjusted survival analyses are shown in **Figure 3**… (overall cohort **N=1,424**).” |  |  |
| Main results | 16 | (a) Give unadjusted estimates and, if applicable, confounder-adjusted estimates and their precision (e.g., 95% confidence interval). Make clear which confounders were adjusted for and why they were included  (b) Report category boundaries when continuous variables were categorized  (c) If relevant, consider translating estimates of relative risk into absolute risk for a meaningful time period | (a) “For prolonged LOS (>15 days)… cachexia… **pooled aRR 0.64, 95% CI 0.51–0.80**…”  (b) “Sensitivity analyses included… **BMI categories (underweight [reference] vs. overweight/obese combined)**…”  (c) N/A |  |  |
| Other analyses | 17 | Report other analyses done—e.g., analyses of subgroups and interactions, and sensitivity analyses | “In exploratory analyses, the association between cachexia and TO **varied by BMI** (**interaction P=0.047**; Figure 1). **Sensitivity analysis** excluding LOS from TO…” |  |  |
| **Discussion** | | | | | |
| Key results | 18 | Summarise key results with reference to study objectives | “This study found no evidence of a ‘double jeopardy’ effect where cachexia combined with NAT worsens outcomes. While cachexia was linked to a higher probability of achieving the composite Textbook Outcome, this was driven by a shorter length of stay, not fewer complications. After multivariable adjustment, cachexia was not independently associated with long-term survival.” |  |  |
| Limitations | 19 | Discuss limitations of the study, taking into account sources of potential bias or imprecision. Discuss both direction and magnitude of any potential bias | This study has **limitations**… residual confounding… registry granularity… which could influence both selection for surgery and outcomes.” | RECORD 19.1: Discuss the implications of using data that were not created or collected to answer the specific research question(s). Include discussion of misclassification bias, unmeasured confounding, missing data, and changing eligibility over time, as they pertain to the study being reported. | **Discussion (Limitations):** "One inherent limitation of a registry-based cohort is the lack of granular data... definition of cachexia relied on patient-reported weight loss... registry lacks detailed data on specific neoadjuvant therapies." |
| Interpretation | 20 | Give a cautious overall interpretation of results considering objectives, limitations, multiplicity of analyses, results from similar studies, and other relevant evidence | “The current study **does not support** the notion that NAT is linked to an additional short-term burden or long-term survival detriment beyond cachexia… [interpretation in light of prior evidence].” |  |  |
| Generalisability | 21 | Discuss the generalisability (external validity) of the study results | “A key strength is the **nationwide** coverage, **enhancing generalizability** to a real-world surgical setting.” |  |  |
| **Other Information** | | | | | |
| Funding | 22 | Give the source of funding and the role of the funders for the present study and, if applicable, for the original study on which the present article is based | Funded in part by **Helse Vest #F-12625** (Norwegian Regional Health Authority Western Region) and **Folke Hermans Fund for Cancer Research**.” |  |  |
| Accessibility of protocol, raw data, and programming code |  | .. |  | RECORD 22.1: Authors should provide information on how to access any supplemental information such as the study protocol, raw data, or programming code. | **Data Availability Statement:** "De-identified registry data are available upon formal application to the NORGAST Registry through the National Health Data Service (Helsedataservice)." |

*Reference: Benchimol EI, Smeeth L, Guttmann A, Harron K, Moher D, Petersen I, Sørensen HT, von Elm E, Langan SM, the RECORD Working Committee. The REporting of studies Conducted using Observational Routinely-collected health Data (RECORD) Statement. *PLoS Medicine* 2015; in press.

*Checklist is protected under Creative Commons Attribution ([CC BY](http://creativecommons.org/licenses/by/4.0/)) license.
