# Supplementary material for: Combined Impact of Neoadjuvant Therapy and Preoperative Cachexia in Patients Undergoing Pancreatoduodenectomy: Is There a “Double Jeopardy”? A National Cohort Study Investigating the Association with Short- and Long-Term Outcomes
Source: Ann Surg Oncol. 2026 Jan 5;33(4):3563–75. doi: 10.1245/s10434-025-18941-y (PMC12982233; doi:10.1245/s10434-025-18941-y)
Supplement: Supplementary file 2 — Supplementary material 2 [file 10434_2025_18941_MOESM2_ESM.docx]

**Supplementary Materials – Index**

| **Supplementary Tables** |  |
| --- | --- |
|  |  |
| Table S1: Missing Data Comparison | Page 2 |
| Table S2: Other Neoplasia ICD-10 Codes | Page 4 |
| Table S3: Unadjusted Postoperative Outcomes by Cachexia | Page 6 |
| Table S4: Sensitivity Analysis: Length of Stay | Page 7 |
| Table S5: Regression Analysis using MI for Prolonged LOS | Page 9 |
| Table S6: Textbook Outcome: Complete Case Analysis | Page 11 |
| Table S7: Prolonged LOS: Complete Case Analysis | Page 13 |
|  |  |
|  |  |
| **Supplementary Figures** |  |
|  |  |
| Figure S1: STROBE Diagram | Page 15 |
| Figure S2: Multivariable Analysis Using MI for Textbook Outcome | Page 16 |
| Figure S3: Component Analysis Cachexia | Page 18 |
| Figure S4: Forest Plot of Multivariable Cox Regression Model | Page 19 |

**Table S1**. Comparison of Complete Cases vs Cases with Missing Covariates

| **Baseline Characteristic** | **Included in Analysis**  N=1,253^1^ | **Excluded (Missing Data)**  N=171^1^ | **N (Excluded)** | ***P***^2^ | **Overall**  N=1,424^1^ |
| --- | --- | --- | --- | --- | --- |
| **Age (years)** | 70.0 (61.1, 74.9) | 68.5 (62.1, 74.9) | 171 | 0.718 | 69.8 (61.3, 74.9) |
| **Gender** |  |  | 171 | 0.672 |  |
| Female | 572 (45.7%) | 81 (47.4%) |  |  | 653 (45.9%) |
| Male | 681 (54.3%) | 90 (52.6%) |  |  | 771 (54.1%) |
| **WHO BMI categories** |  |  | 151 | 0.662 |  |
| Normal | 606 (48.4%) | 71 (47.0%) |  |  | 677 (48.2%) |
| Underweight | 45 (3.6%) | 6 (4.0%) |  |  | 51 (3.6%) |
| Overweight | 446 (35.6%) | 50 (33.1%) |  |  | 496 (35.3%) |
| Obese | 156 (12.5%) | 24 (15.9%) |  |  | 180 (12.8%) |
| Missing | 0 | 20 |  |  | 20 |
| **Preoperative Diagnosis** |  |  | 171 | **0.004** |  |
| Malignancy | 1,159 (92.5%) | 147 (86.0%) |  |  | 1,306 (91.7%) |
| Neoplasia | 94 (7.5%) | 24 (14.0%) |  |  | 118 (8.3%) |
| **ASA score** |  |  | 171 | 0.224 |  |
| 1-2 | 619 (49.4%) | 76 (44.4%) |  |  | 695 (48.8%) |
| ≥3 | 634 (50.6%) | 95 (55.6%) |  |  | 729 (51.2%) |
| **WHO ECOG score** |  |  | 132 | 0.789 |  |
| 0-1 | 1,175 (93.8%) | 123 (93.2%) |  |  | 1,298 (93.7%) |
| ≥2 | 78 (6.2%) | 9 (6.8%) |  |  | 87 (6.3%) |
| Missing | 0 | 39 |  |  | 39 |
| **Neoadjuvant therapy** |  |  | 171 | 0.479 |  |
| No | 1,074 (85.7%) | 150 (87.7%) |  |  | 1,224 (86.0%) |
| Yes | 179 (14.3%) | 21 (12.3%) |  |  | 200 (14.0%) |
| **Diabetes mellitus** |  |  | 171 | 0.439 |  |
| No | 1,041 (83.1%) | 138 (80.7%) |  |  | 1,179 (82.8%) |
| Yes | 212 (16.9%) | 33 (19.3%) |  |  | 245 (17.2%) |
| **Heart disease** |  |  | 171 | **0.002** |  |
| No | 1,229 (98.1%) | 160 (93.6%) |  |  | 1,389 (97.5%) |
| Yes | 24 (1.9%) | 11 (6.4%) |  |  | 35 (2.5%) |
| **Lung disease** |  |  | 171 | 0.073 |  |
| No | 1,231 (98.2%) | 164 (95.9%) |  |  | 1,395 (98.0%) |
| Yes | 22 (1.8%) | 7 (4.1%) |  |  | 29 (2.0%) |
| **Albumin (g/L)** | 40.0 (36.0, 43.0) | 40.5 (38.0, 43.0) | 126 | 0.541 | 40.0 (36.0, 43.0) |
| Missing | 0 | 45 |  |  | 45 |
| **CRP (mg/L)** | 5.0 (2.0, 13.0) | 11.0 (5.0, 23.5) | 64 | **<0.001** | 5.0 (2.0, 13.0) |
| Missing | 0 | 107 |  |  | 107 |
| **Preoperative Cachexia** |  |  | 171 | **<0.001** |  |
| No cachexia | 702 (56.0%) | 134 (78.4%) |  |  | 836 (58.7%) |
| Cachexia | 551 (44.0%) | 37 (21.6%) |  |  | 588 (41.3%) |
| ^1^Median (Q1, Q3); n (%) | | | | | |
| ^2^Wilcoxon rank sum test; Pearson's Chi-squared test; Fisher's exact test | | | | | |
| **Legend**: N columns show non-missing observations for each variable within each stratum. Percentages are among non-missing observations; p-values use available-case data. | | | | | |
| **Abbreviations**: ASA, American Society of Anesthesiologists; BMI, Body Mass Index; CRP, C-reactive Protein; ECOG, Eastern Cooperative Oncology Group; WHO, World Health Organization. | | | | | |

**Table S2.** Composition of the “Other Neoplasia” Category: Frequency of Specific ICD-10 D-codes by Preoperative Cachexia Status

|  | **Preoperative Cachexia Status** | |
| --- | --- | --- |
| **Characteristic** | **No cachexia**  N=96^1^ | **Cachexia**  N=22^1^ |
| **Specific ICD-10 Code (D-Group)** |  |  |
| D136 Benign neoplasm of pancreas, other and unspecified parts | 31 (32%) | 6 (27%) |
| D377 Neoplasm of uncertain or unknown behaviour of other digestive organs | 17 (18%) | 4 (18%) |
| D132 Benign neoplasm of extrahepatic bile ducts | 18 (19%) | 2 (9.1%) |
| D3771 Tumor with clinically uncertain malignant potential in other specified digestive organ | 12 (13%) | 4 (18%) |
| D135 Benign neoplasm of ampulla of Vater | 3 (3.1%) | 3 (14%) |
| D376 Neoplasm of uncertain or unknown behaviour of liver, gallbladder and bile ducts | 1 (1.0%) | 2 (9.1%) |
| D014 Carcinoma in situ of other and unspecified parts of intestine | 2 (2.1%) | 0 (0%) |
| D015 Carcinoma in situ of liver, gallbladder and bile ducts | 1 (1.0%) | 1 (4.5%) |
| D3761 Tumor with clinically uncertain malignant potential in liver, gallbladder, or bile duct | 2 (2.1%) | 0 (0%) |
| D3770 Tumor with pathologically anatomically proven uncertain malignant potential in other specified digestive organ | 2 (2.1%) | 0 (0%) |
| D379 Neoplasm of uncertain or unknown behaviour of digestive organ, unspecified | 2 (2.1%) | 0 (0%) |
| D137 Benign neoplasm of endocrine pancreas | 1 (1.0%) | 0 (0%) |
| D357 Neoplasm of uncertain or unknown behaviour of other specified endocrine glands | 1 (1.0%) | 0 (0%) |
| D3711 Tumor with clinically uncertain malignant potential in stomach | 1 (1.0%) | 0 (0%) |
| D372 Neoplasm of uncertain or unknown behaviour of small intestine | 1 (1.0%) | 0 (0%) |
| D449 Neoplasm of uncertain or unknown behaviour of endocrine gland, unspecified | 1 (1.0%) | 0 (0%) |
| ^1^n (%) | | |

**Table S3.** Unadjusted Postoperative Outcomes Following Pancreatoduodenectomy, Stratified by Preoperative Cachexia Status

| **Characteristic** | **Overall**  N=1,424^1^ | **No cachexia**  N=836^1^ | **Cachexia**  N=588^1^ | ***P*-value**^2^ |
| --- | --- | --- | --- | --- |
| **Textbook Outcome (Yes)** | 650 (45.6%) | 344 (41.1%) | 306 (52.0%) | **<0.001** |
| **Length of stay (days)** | 9.0 (7.0, 15.0) | 10.0 (7.0, 16.0) | 8.0 (6.0, 13.0) | **<0.001** |
| **Accordion score (grade)** |  |  |  | 0.149 |
| Death (6) | 23 (1.6%) | 16 (1.9%) | 7 (1.2%) |  |
| Major (3-5) | 456 (32.0%) | 281 (33.6%) | 175 (29.8%) |  |
| Minor (1-2) | 945 (66.4%) | 539 (64.5%) | 406 (69.0%) |  |
| **Readmission** | 169 (12.9%) | 103 (13.4%) | 66 (12.1%) | 0.490 |
| **Readmission Other** | 131 (10.0%) | 79 (10.3%) | 52 (9.6%) | 0.659 |
| **Relaparotomy** | 176 (12.4%) | 96 (11.5%) | 80 (13.6%) | 0.231 |
| **Relaparotomy Readmission** | 37 (14.0%) | 16 (9.8%) | 21 (20.6%) | **0.014** |
| **30-day mortality** | 21 (1.5%) | 14 (1.7%) | 7 (1.2%) | 0.455 |
| **90-day mortality** | 36 (2.5%) | 20 (2.4%) | 16 (2.7%) | 0.697 |
| ^1^n (%); Median (Q1, Q3) | | | | |
| ^2^Pearson's Chi-squared test; Wilcoxon rank sum test | | | | |
| **Legend**: Textbook Outcome defined as absence of Accordion grade ≥3 complication, prolonged length of stay (>15 days), reoperation, unplanned 30-day readmission, and 30-day/in-hospital mortality. Accordion score grade represents the most severe postoperative complication grade (Grade 4-5 considered major, Grade 6 death). Relaparotomy Readmission indicates relaparotomy during an unplanned readmission. | | | | |

**Table S4.** Sensitivity Analysis: Multivariable Model for Modified Textbook Outcome (Excluding Prolonged Length of Stay)

| **Variable** | **aRR (95% CI)** | ***P*** |
| --- | --- | --- |
| **Primary exposures** |  |  |
| Cachexia: Yes (Ref: No) | 1.13 (1.03–1.25) | **0.013** |
| Neoadjuvant therapy: Yes (Ref: No) | 1.03 (0.85–1.23) | 0.791 |
| Cachexia × NAT: P for interaction | — | 0.215 |
| **Demographics** |  |  |
| Age (per 10 years) | 1.00 (0.96–1.05) | 0.882 |
| Sex: Male (Ref: Female) | 0.80 (0.74–0.88) | **<0.001** |
| **Anthropometrics** |  |  |
| BMI: Normal (Ref) | 1.00 (Ref) | — |
| BMI: Underweight | 0.97 (0.78–1.20) | 0.780 |
| BMI: Overweight | 0.90 (0.81–0.99) | **0.034** |
| BMI: Obese | 0.93 (0.80–1.07) | 0.293 |
| **Comorbidity** |  |  |
| Diabetes: Yes (Ref: No) | 1.07 (0.95–1.20) | 0.276 |
| Heart disease: Yes (Ref: No) | 1.12 (0.84–1.48) | 0.438 |
| Lung disease: Yes (Ref: No) | 1.00 (0.69–1.46) | 0.991 |
| **Laboratory** |  |  |
| Albumin (g/L) | 1.00 (0.99–1.01) | 0.754 |
| CRP (mg/L) | 1.00 (1.00–1.00) | 0.955 |
| **Disease** |  |  |
| Diagnosis: Other neoplasia (Ref: Malignancy) | 0.80 (0.65–0.98) | **0.034** |
| **Legend**: The modified textbook outcome (excluding length of stay) was defined as the absence of severe complications (Accordion score ≥3), reoperation, unplanned 30-day readmission, or 30-day/in-hospital mortality following pancreatoduodenectomy. Table shows pooled adjusted risk ratios (aRR) with 95% confidence intervals (CI) and *P*-values from modified Poisson regression with robust standard errors applied to 20 multiply imputed datasets (N=1,424). The model was adjusted for all variables listed in the table. | | |
| **Abbreviations**: aRR, adjusted Risk Ratio; ASA, American Society of Anesthesiologists; BMI, Body Mass Index; CI, Confidence Interval; CRP, C-reactive Protein; ECOG, Eastern Cooperative Oncology Group; NAT, Neoadjuvant Therapy; WHO, World Health Organization. | | |

**Table S5.** Multivariable Poisson Regression Analysis for Factors Associated with Prolonged Length of Stay

| **Variable** | **aRR (95% CI)** | ***P*** |
| --- | --- | --- |
| **Primary exposures** |  |  |
| Cachexia: Yes (Ref: No) | 0.64 (0.51–0.80) | **<0.001** |
| Neoadjuvant therapy: Yes (Ref: No) | 0.72 (0.47–1.13) | 0.153 |
| Cachexia × NAT: P for interaction | — | 0.638 |
| **Demographics** |  |  |
| Age (per 10 years) | 1.11 (0.99–1.24) | 0.084 |
| Sex: Male (Ref: Female) | 1.41 (1.16–1.72) | **<0.001** |
| **Anthropometrics** |  |  |
| BMI: Normal (Ref) | 1.00 (Ref) | — |
| BMI: Underweight | 1.35 (0.80–2.29) | 0.259 |
| BMI: Overweight | 1.12 (0.90–1.38) | 0.310 |
| BMI: Obese | 1.40 (1.06–1.84) | **0.017** |
| **Comorbidity** |  |  |
| Diabetes: Yes (Ref: No) | 0.90 (0.69–1.16) | 0.414 |
| Heart disease: Yes (Ref: No) | 1.13 (0.67–1.91) | 0.644 |
| Lung disease: Yes (Ref: No) | 0.99 (0.50–1.95) | 0.970 |
| **Laboratory** |  |  |
| Albumin (g/L) | 0.99 (0.97–1.01) | 0.340 |
| CRP (mg/L) | 1.00 (0.99–1.00) | 0.384 |
| **Disease** |  |  |
| Diagnosis: Other neoplasia (Ref: Malignancy) | 1.62 (1.24–2.11) | **<0.001** |
| **Legend**: Prolonged length of stay (LOS) was defined as postoperative LOS greater than the 75th percentile (>15 days). Table shows pooled adjusted risk ratios (aRR) with 95% confidence intervals (CI) and *P*-values from modified Poisson regression with robust standard errors applied to 20 multiply imputed datasets (N=1,424). The model was adjusted for all variables listed in the table. | | |
| **Abbreviations**: aRR, adjusted Risk Ratio; ASA, American Society of Anesthesiologists; BMI, Body Mass Index; CI, Confidence Interval; CRP, C-reactive Protein; ECOG, Eastern Cooperative Oncology Group; NAT, Neoadjuvant Therapy; WHO, World Health Organization. | | |

**Table S6**. Complete Case Analysis: Multivariable Poisson Regression Analysis of Factors Associated with Achieving the Textbook Outcome (N=1,253)

| **Variable** | **aRR (95% CI)** | ***P*** |
| --- | --- | --- |
| **Primary exposures** |  |  |
| Cachexia: Yes (Ref: No) | 1.30 (1.14–1.49) | **<0.001** |
| Neoadjuvant therapy: Yes (Ref: No) | 1.21 (0.96–1.53) | 0.114 |
| Cachexia × NAT: P for interaction | — | 0.131 |
| **Demographics** |  |  |
| Age (per 10 years) | 1.00 (0.94–1.06) | 0.905 |
| Sex: Male (Ref: Female) | 0.76 (0.67–0.85) | **<0.001** |
| **Anthropometrics** |  |  |
| BMI: Normal (Ref) | 1.00 (Ref) | — |
| BMI: Underweight | 0.97 (0.75–1.27) | 0.846 |
| BMI: Overweight | 0.85 (0.74–0.97) | **0.016** |
| BMI: Obese | 0.92 (0.76–1.11) | 0.361 |
| **Comorbidity** |  |  |
| Diabetes: Yes (Ref: No) | 1.16 (0.99–1.34) | 0.059 |
| Heart disease: Yes (Ref: No) | 0.96 (0.57–1.63) | 0.884 |
| Lung disease: Yes (Ref: No) | 0.75 (0.39–1.45) | 0.393 |
| **Laboratory** |  |  |
| Albumin (g/L) | 1.00 (0.98–1.01) | 0.574 |
| CRP (mg/L) | 1.00 (1.00–1.00) | 0.651 |
| **Disease** |  |  |
| Diagnosis: Other neoplasia (Ref: Malignancy) | 0.71 (0.52–0.96) | **0.028** |

**Legend**: aRR > 1 indicates increased probability of achieving the textbook outcome; aRR < 1 indicates decreased probability. Estimates are from modified Poisson regression with robust standard errors using a complete-case analysis. The model was adjusted for all variables listed in the table.

**Abbreviations:** aRR, adjusted Risk Ratio; ASA, American Society of Anesthesiologists; BMI, Body Mass Index; CI, Confidence Interval; CRP, C-reactive Protein; ECOG, Eastern Cooperative Oncology Group; NAT, Neoadjuvant Therapy; WHO, World Health Organization.

**Table S7**. Complete Case Analysis: Multivariable Poisson Regression Analysis of Factors Associated with Prolonged Length of Stay

| **Variable** | **Adjusted RR (95% CI)** | ***P*** |
| --- | --- | --- |
| **Primary exposures** |  |  |
| Cachexia: Yes (Ref: No) | 0.61 (0.48–0.78) | **<0.001** |
| Neoadjuvant therapy: Yes (Ref: No) | 0.71 (0.43–1.16) | 0.174 |
| Cachexia × NAT: P for interaction | — | 0.414 |
| **Demographics** |  |  |
| Age (years) | 1.01 (1.00–1.02) | 0.173 |
| Sex: Male (Ref: Female) | 1.52 (1.23–1.88) | **<0.001** |
| **Anthropometrics** |  |  |
| BMI: Normal (Ref) | 1.00 (Ref) | — |
| BMI: Underweight | 1.33 (0.76–2.34) | 0.321 |
| BMI: Overweight | 1.14 (0.91–1.44) | 0.243 |
| BMI: Obese | 1.48 (1.11–1.98) | **0.007** |
| **Comorbidity** |  |  |
| Diabetes: Yes (Ref: No) | 0.88 (0.66–1.16) | 0.363 |
| Heart disease: Yes (Ref: No) | 0.96 (0.44–2.06) | 0.907 |
| Lung disease: Yes (Ref: No) | 1.22 (0.60–2.47) | 0.580 |
| **Laboratory** |  |  |
| Albumin (g/L) | 0.99 (0.97–1.01) | 0.367 |
| CRP (mg/L) | 1.00 (0.99–1.00) | 0.266 |
| **Disease** |  |  |
| Diagnosis: Other neoplasia (Ref: Malignancy) | 1.68 (1.26–2.23) | **<0.001** |
| **Legend**: Prolonged length of stay (LOS) was defined as postoperative LOS greater than the 75th percentile (> 15 days). Table shows adjusted risk ratios (aRR) with 95% confidence intervals (CI) and *P*-values from modified Poisson regression with robust standard errors applied to complete cases (N=1253; 171 excluded from total N=1424). The model was adjusted for all variables listed in the table. Overall prolonged LOS rate among complete cases: 23.4% (293/1253).  **Abbreviations**: aRR, adjusted Risk Ratio; ASA, American Society of Anesthesiologists; BMI, Body Mass Index; CI, Confidence Interval; CRP, C-reactive Protein; ECOG, Eastern Cooperative Oncology Group; NAT, Neoadjuvant Therapy; WHO, World Health Organization. | | |

**Figure S1**. STROBE Diagram


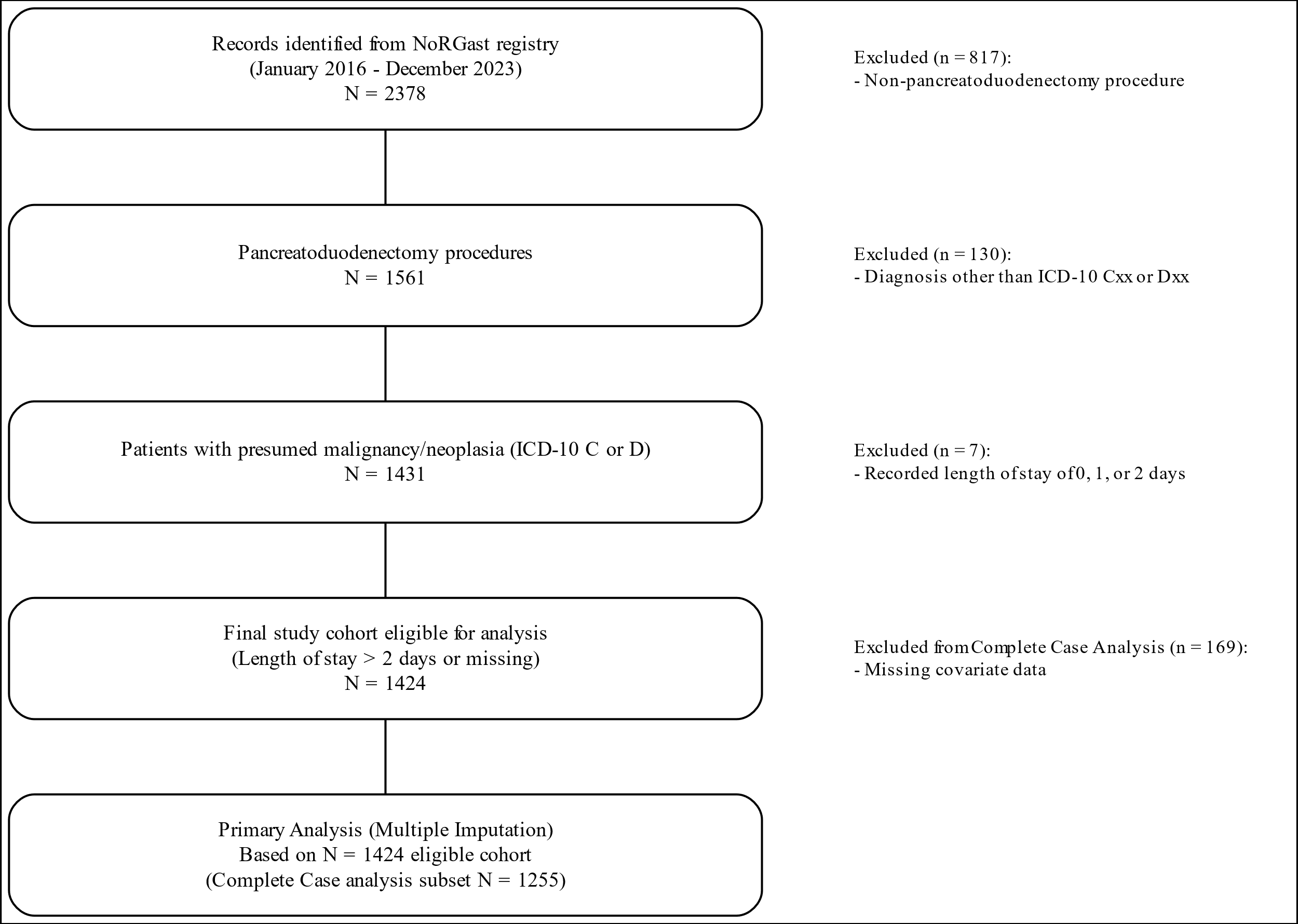


**Figure S2.** Multivariable Analysis using Multiple Imputation for Factors Associated with Achieving the Textbook Outcome

**
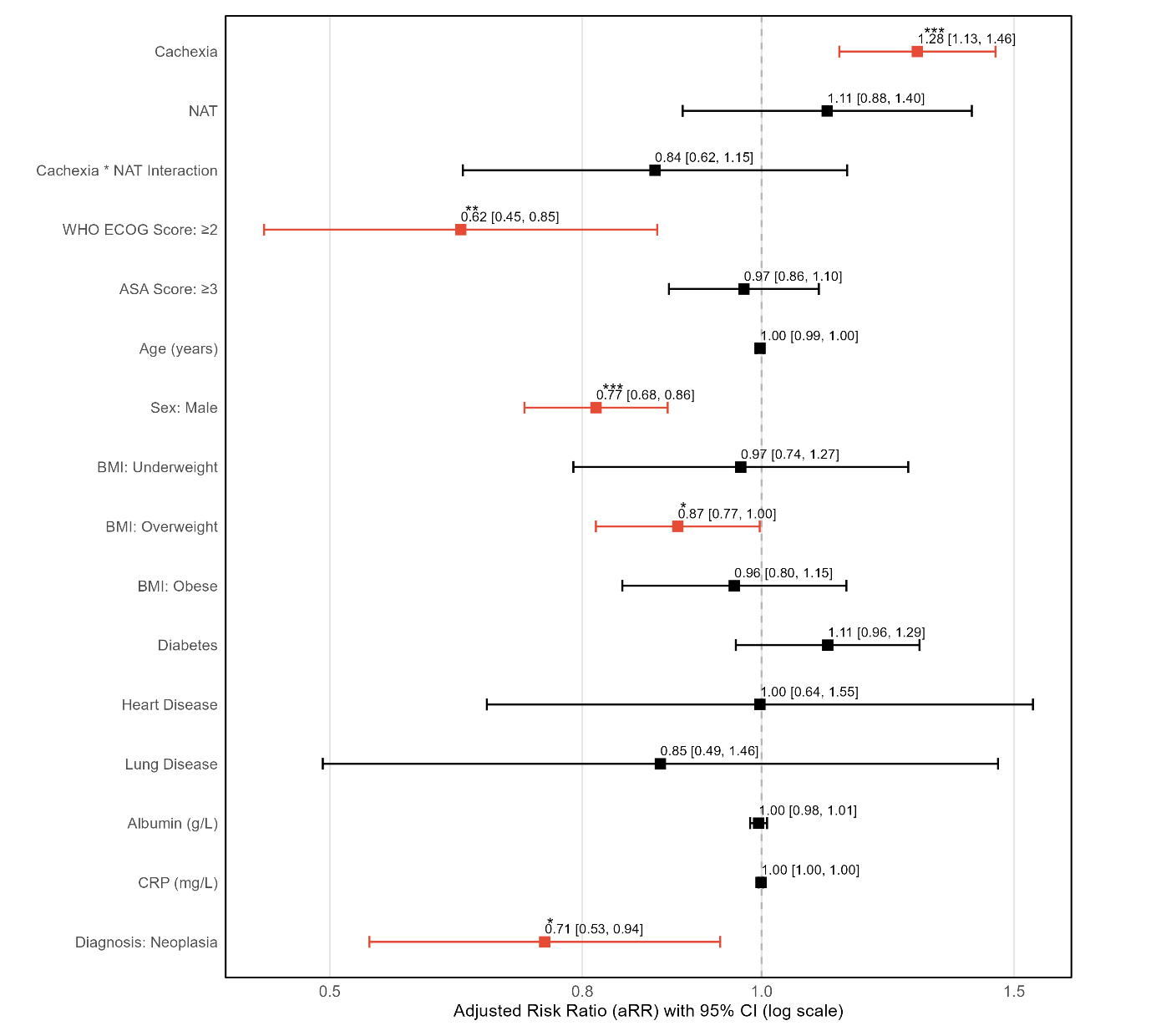
**

**Legend**: Forest plot displaying pooled adjusted risk ratios (aRRs) and 95% confidence intervals (CIs) from the multivariable Poisson regression model using multiple imputation (m=20) for achieving the composite textbook outcome after pancreatoduodenectomy (Total N=1424). Dots represent the pooled aRR point estimate, and horizontal lines represent the pooled 95% CI. The vertical dashed line indicates an aRR of 1.0 (no association). Reference groups include no cachexia, no neoadjuvant therapy (NAT), female sex, normal BMI, ASA score 1-2, WHO ECOG score 0-1, no diabetes, no heart disease, no lung disease, and malignancy diagnosis. Points and confidence intervals are colored red if the association is statistically significant (p<0.05) and black otherwise. Model adjusted for all variables shown. Significance levels derived from pooled results: * p<0.05, ** p<0.01, *** p<0.001.

**Figure S3**. Adjusted Effect of Preoperative Cachexia on Individual Short-Term Outcome Components (Excluding Length of Stay)


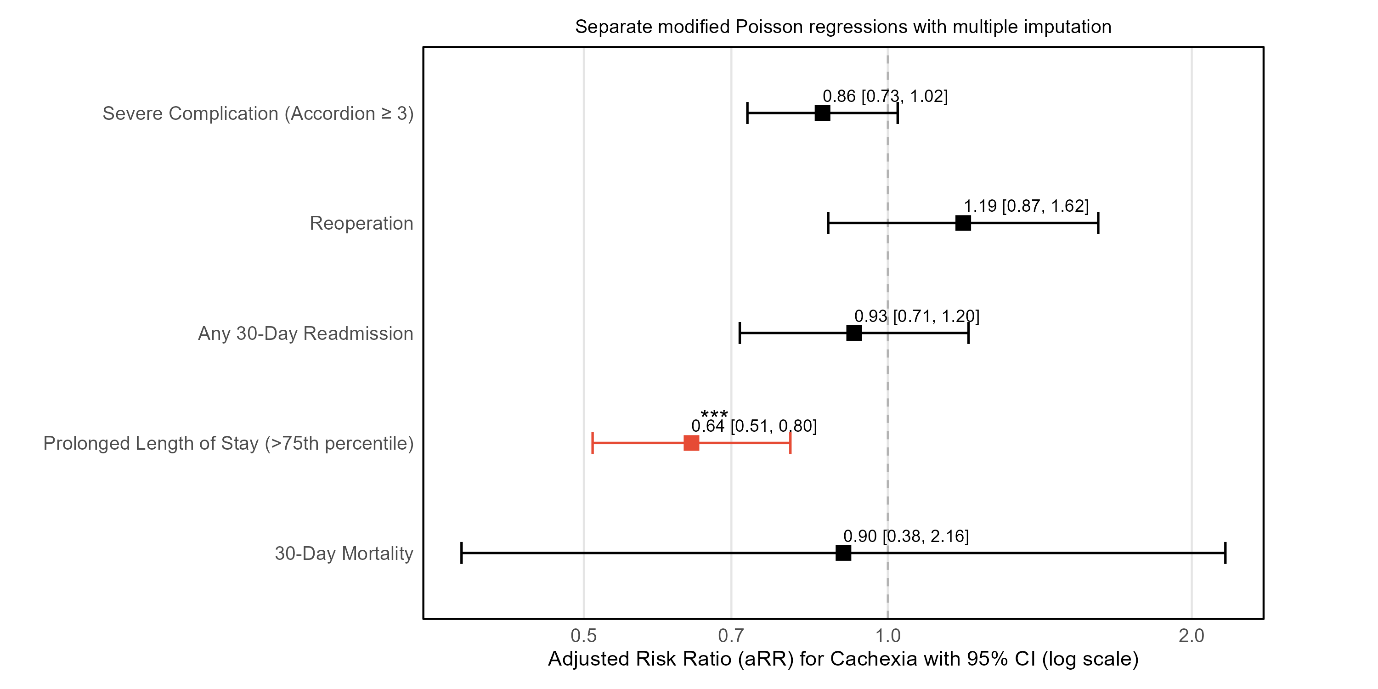


**Legend:** Forest plot displays pooled adjusted risk ratios (aRR) with 95% confidence intervals (CI) for the association between preoperative cachexia (reference: no cachexia) and individual short-term outcome components after pancreatoduodenectomy: Severe complication (Accordion ≥3), reoperation, any 30-day readmission, prolonged length of stay (>75th percentile), and 30-day mortality. Estimates are from separate modified Poisson regressions (log link, robust standard errors) fit to 20 multiply imputed datasets (N = 1,424) and adjusted for the same covariates as the primary textbook-outcome model (Methods/Table 2). The dashed vertical line indicates aRR = 1.0 (no association). The x-axis is log-scaled.

**Figure S4**. Forest Plot of Multivariable Cox Proportional-Hazards Regression Analysis for Long-Term Overall Survival


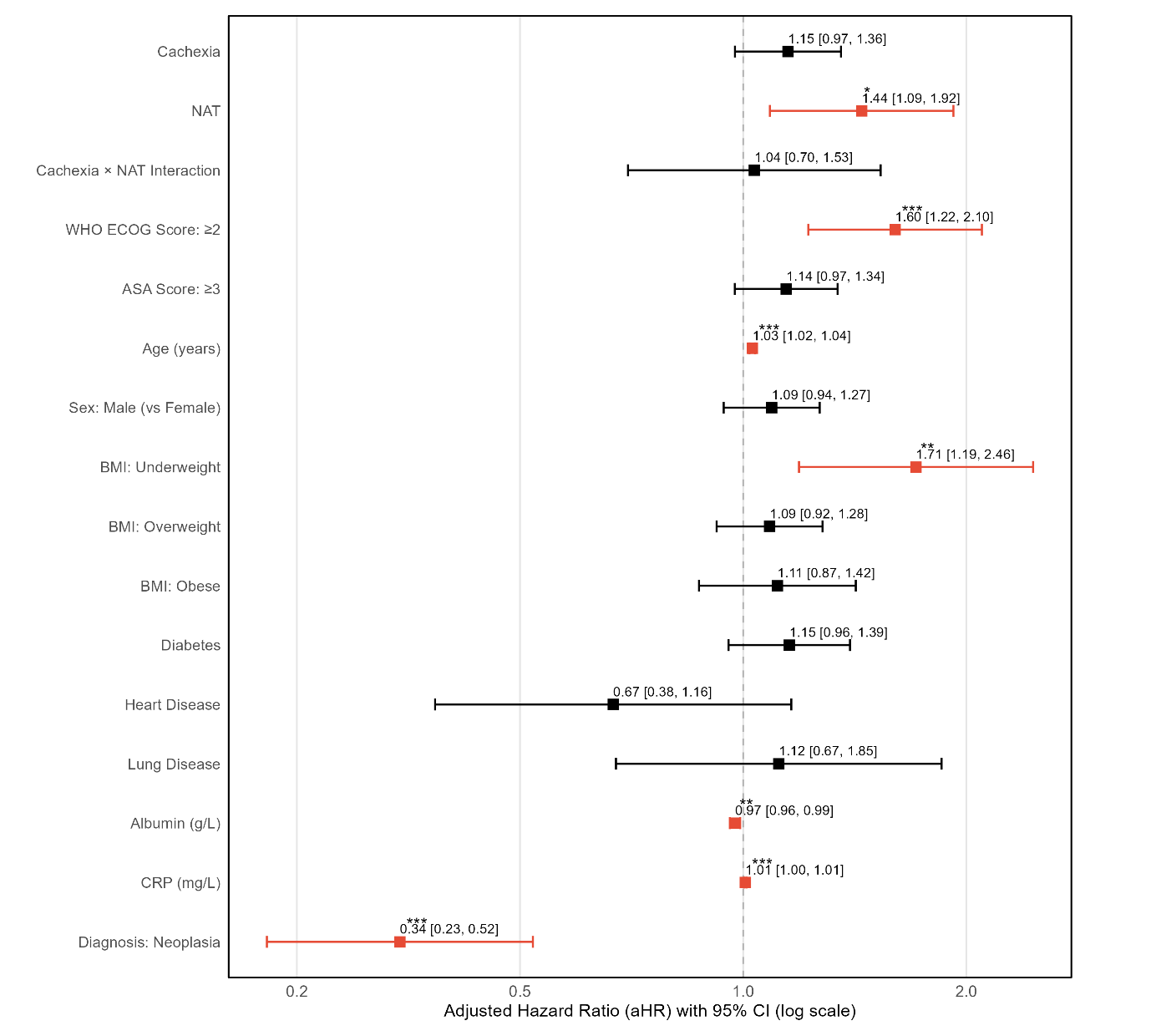


**Legend**: Forest plot displaying adjusted Hazard Ratios (aHRs) and 95% Confidence Intervals (CIs) from the multivariable Cox proportional-hazards regression model for long-term overall survival using multiply imputed datasets (N=1,424). The model included preoperative cachexia, neoadjuvant therapy (NAT), their interaction term (Cachexia × NAT), and was adjusted for age, sex, BMI category, ASA score, WHO/ECOG score, diabetes mellitus, preoperative albumin, preoperative C-reactive protein, and preoperative diagnosis type. Reference groups include no cachexia, no neoadjuvant therapy (NAT), female sex, normal BMI, ASA score 1-2, WHO ECOG score 0-1, no diabetes, no heart disease, no lung disease, and malignancy diagnosis. Points and confidence intervals are colored red if the association is statistically significant (p<0.05) and black otherwise. Model adjusted for all variables shown. Significance levels derived from pooled results: * p<0.05, ** p<0.01, *** p<0.001.
